# GIT-Net: An Ensemble Deep Learning-Based GI Tract Classification of Endoscopic Images

**DOI:** 10.3390/bioengineering10070809

**Published:** 2023-07-05

**Authors:** Hemalatha Gunasekaran, Krishnamoorthi Ramalakshmi, Deepa Kanmani Swaminathan, Andrew J, Manuel Mazzara

**Affiliations:** 1 Information Technology, University of Technology and Applied Sciences, Ibri 516, Oman; hemalatha.ibr@cas.edu.om; 2 Information Technology, Alliance College of Engineering and Design, Alliance University, Bengaluru 562106, India; ramalakshmi.k@alliance.edu.in; 3 Information Technology, Sri Krishna College of Engineering and Technology, Coimbatore 641008, India; deepakanmanis@skcet.ac.in; 4 Computer Science and Engineering, Manipal Institute of Technology, Manipal Academy of Higher Education, Manipal 576104, India; 5 Institute of Software Development and Engineering, Innopolis University, 420500 Innopolis, Russia; m.mazzara@innopolis.ru

**Keywords:** ensemble learning, base learners, gastrointestinal tract, deep learning, transfer learning

## Abstract

This paper presents an ensemble of pre-trained models for the accurate classification of endoscopic images associated with Gastrointestinal (GI) diseases and illnesses. In this paper, we propose a weighted average ensemble model called GIT-NET to classify GI-tract diseases. We evaluated the model on a KVASIR v2 dataset with eight classes. When individual models are used for classification, they are often prone to misclassification since they may not be able to learn the characteristics of all the classes adequately. This is due to the fact that each model may learn the characteristics of specific classes more efficiently than the other classes. We propose an ensemble model that leverages the predictions of three pre-trained models, DenseNet201, InceptionV3, and ResNet50 with accuracies of 94.54%, 88.38%, and 90.58%, respectively. The predictions of the base learners are combined using two methods: model averaging and weighted averaging. The performances of the models are evaluated, and the model averaging ensemble has an accuracy of 92.96% whereas the weighted average ensemble has an accuracy of 95.00%. The weighted average ensemble outperforms the model average ensemble and all individual models. The results from the evaluation demonstrate that utilizing an ensemble of base learners can successfully classify features that were incorrectly learned by individual base learners.

## 1. Introduction

A huge number of people in the world are affected by a potentially fatal gastrointestinal disorder (GID). According to the WHO (World Health Organization), 1.8 million people worldwide die from digestive diseases each year [1]. In India, around 18% of the population suffers from GI tract disorders [2]. Gastrointestinal disorders are digestive tract (DT) disorders that may lead to gastrointestinal cancer. The disease can be diagnosed by physically examining the endoscopy images of the GI tract or by performing some sophisticated laboratory test or through a radiographic procedure. Early detection of this disease can radically reduce the death rate. The endoscopy images [3] obtained during the physical examination play an important role in disease identification. Computer-aided diagnosis (CAD) is a technology that uses artificial intelligence and medical image processing to help the radiologist to interpret the images and aid in disease diagnosis. CAD helps the radiologist to identify abnormalities and take decisions much faster. Initially, machine learning techniques such as Naïve Bayes, Decision Tree (DT), Random Forest, Support Vector Machine (SVM), etc., are used to classify the endoscopic images [4,5,6]. The performance of the machine learning model mainly depends on the features identified to develop the model. The primary limitation of a machine-learning model is that it requires a domain expert, such as a gastroenterologist, to correctly identify the important features used for classification. Due to the recent advancements in AI, Deep Learning algorithms [7,8,9,10] play a vital role in assisting radiologists during physical examination and aiding in the diagnosis of disease. Deep learning techniques are capable of automatic feature extraction that contributes to improving the performance of the model. Convolutional neural network (CNN) shows better performance in feature extraction than machine-learning models [9]. The accuracy of the prediction is determined by the model architecture, the hyper-parameter of the model, and the quality and size of the dataset. The major limitation of the CNN model is that it requires a large amount of data to build a robust model. Unfortunately, in the medical field, the amount of training and test data available to build a robust model is limited. In such a scenario, transfer learning techniques play a vital role in building a robust model.

In transfer learning, the pre-trained model can be used either as a feature extractor or directly for the classification of the target dataset. Using a pre-trained model is very efficient as the model need not be trained from scratch, and as a result, computing costs and training time can be saved. If the source dataset and the target dataset are completely different, then the pre-trained model can be fine-tuned by adding a few layers on top of the base layers to learn the specific features of the dataset.

The existing methods use individual pre-trained models to classify the GI tract disorder. These models’ performances mainly depend on the type of pre-trained model used, while an ensemble model can combine several base models to capture classifiers with greater accuracy. Additionally, an ensemble model can address the over-fitting problem more effectively, as it works on multiple parameters of different models at the same time and can thus effectively reduce variance.

In this paper, three pre-trained models, ResNet50 (Residual Network), DenseNet201 (Densely Connected Convolutional Networks), and Inception v3 are trained on the given dataset. We discovered that each model predicts a subset of classes better than the other models. Instead of training a single model, multiple models can be trained, and predictions can be combined to avoid the variances of the neural network model.

The ensemble approach combines multiple weak learners/base learners to create a strong learner. Because each base learner has a unique architecture, they learn different patterns from the same data. Patterns incorrectly learned by one model can be correctly classified by another model, giving each model a distinct perspective on the same data. As a result, combining predictions from multiple models may result in better accuracy and predictions than the individual models.

The main contributions of this paper include:

- We propose a deep ensemble model with three fine-tuned base learners, namely ResNet50, DenseNet201, and InceptionV3.
- The proposed approach is evaluated on the KVASIR v2 dataset, consisting of eight classes with 8000 samples.
- We conducted extensive experiments to show significant improvement in accuracy, precision, and recall of the ensemble model compared to the baseline models.

The proposed weighted average ensemble model of DenseNet201, InceptionV3, and ResNet50 attained an accuracy of 95%. The accuracy of the model can be improved further by training the pre-trained model from scratch. However, this may result in higher training time; to avoid this, the first ten layers of the pre-trained models were frozen and the weights from the ImageNet dataset were used without modification.

The remainder of this paper is organized as follows: Section 2 presents related works in the field of GI diagnosis; Section 3 describes the dataset used for this study; Section 4 outlines the architecture of the pre-trained models; Section 5 outlines the architecture of the proposed ensemble model; Section 6 presents the findings derived from the proposed method; Section 7 contains discussion; and finally, Section 8 provides a conclusion.

## 2. Literature Review

Melaku et al. [11] extracted the features of endoscopy images using pre-trained models such as VGGNet and InceptionV3 on the Hyper KVASIR dataset with 23 classes. To concatenate and categorize the collected features, machine learning classification techniques such as SVM, Softmax, k-Nearest Neighbor, and Random Forest were utilized. SVM achieved the highest accuracy of 98% when compared to other classification techniques. M Hmoud et al. [1] used pre-trained models such as GoogLeNet, ResNet-50, and AlexNet to classify endoscopy images. The authors used a KVASIR dataset with five classes. Among the three pre-trained models, AlexNet achieved an accuracy of 97%, a sensitivity of 96.8%, and AUC (Area Under Curve) of 99.98%. Yogapriya et al. [12] classified GI tract diseases using pre-trained models such as VGG16, ResNet-18, and GoogLeNet. The authors used a KVASIR v2 dataset with 6702 images of eight classes. The VGG16 model achieved the highest accuracy of around 96.33% when compared with other models. Zenebe et al. [13] used special factors for the classification of GI tract diseases. The authors proposed a deep CNN-based spatial attention mechanism with encoder and decoder layers for classification. The dataset included 12,147 GI images. The attention mechanism had a higher accuracy of around 92.84% when compared to other models such as ResNet, GoogLeNet, and DenseNet. The authors also employed t-distributed stochastic neighbor embedding (t-SNE) and a confusion matrix for both visualizing the results and validating the performance.

Ahmen Khan et al. [14] presented a solution for the segmentation of ulcers and classification of gastrointestinal infections by utilizing Mask-RCNN and deep CNN feature optimization. The authors used the pre-trained CNN model ResNet101 for feature detection. The features were first optimized using the grasshopper optimization method. The best-selected features were then used in a multi-class support vector machine (MSVM) for final classification. The accuracy of this classification approach was 99.13%. Zhou et al. [15] employed a combination of deep CNNs and ensemble learning to classify Biliary atresia (BA) using sonographic gallbladder images. Five different CNN models were used, and their output predictions were averaged for predicting class labels. Each CNN was trained on a different set of training samples using five-fold cross-validation.

Mohammad et al. [16] employed pre-trained models such as InceptionV3 and DenseNet-201 for deep feature extraction. Subsequently, they fused and optimized these features with a modified dragonfly optimization method. Lastly, they used a machine learning algorithm for classification with an accuracy of 99.8% on a stomach diseases dataset. In their comparison of pre-trained models, Escobar et al. [17] found that VGG-19 achieved an accuracy of 98.20%, outperforming others such as DenseNet-201, ResNet-50, Xception, and VGG-16.

Gamage et al. [18] proposed an ensemble model consisting of pre-trained DenseNet-201, ResNet-18, and VGG-16 models. These models were used to extract the features from the dataset. The extracted features were combined into a single feature vector and were given as an input to the Global Average Pooling layer followed by the output layer. The authors obtained an accuracy of around 97% when compared to other state-of-the-art models. Shahbaz Ayyaz et al. [19] proposed a hybrid approach to classifying endoscopy images of the stomach. The authors used pre-trained models such as VGG16 and AlexNet to extract the features from the dataset. The extracted features were combined by selecting the best features using a GA (Genetic Algorithm). Finally, the authors used CubicSVM for classification and achieved an accuracy of around 99.8%.

Most of the presented approaches in this section developed a single model and evaluated the overall accuracy of the model. However, when the individual accuracy of the classes is considered, each model predicts a subset of classes more accurately than the other classes. Therefore, we propose a deep ensemble of fine-tuned pre-trained models to classify GI tract endoscopic images.

## 3. Dataset

**Figure 1:**
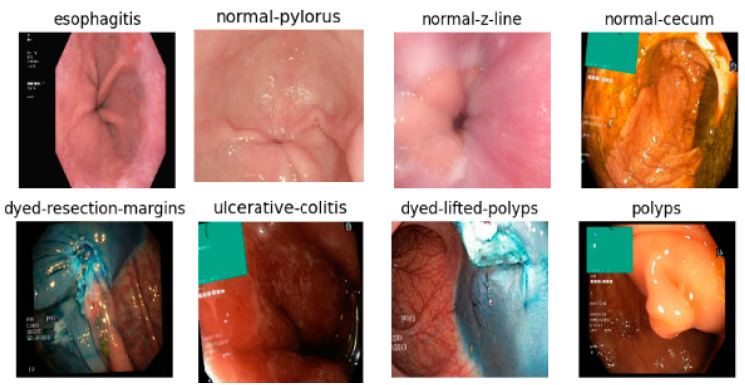
KVASIR v2 Dataset.

In this paper, the proposed ensemble model is tested with a publicly available dataset, namely the KVASIR v2 dataset [5]. The KVASIR v2 dataset is available in Kaggle (https://www.kaggle.com/datasets/plhalvorsen/KVASIR-v2-a-gastrointestinal-tract-dataset) (accessed on 3 January 2023) and it contains 8000 images classified as eight different classes, namely, ‘dyed-lifted-polyps’, ‘dyed-resection-margins’, ‘esophagitis’, ‘normal-cecum’, ‘normal-pylorus’, ‘normal-z-line’, ‘ulcerative-colitis’, and ‘polyps’, as shown in Figure 1. The dataset is about endoscopic images of the GI tract. The samples in each class are equally distributed, with 1000 images per class. Augmenter Pipeline is a set of data augmentation strategies that can be used to generate new and diverse data for deep learning models [20]. These strategies increase the amount of data available for training, as well as create more diverse data that can make the model more accurate and robust. This is done by flipping, rotating, cropping, scaling, adding noise, and changing the color channels to the existing data. By doing so, it can help to reduce overfitting and improve the generalization of the model.

**Figure 2:**
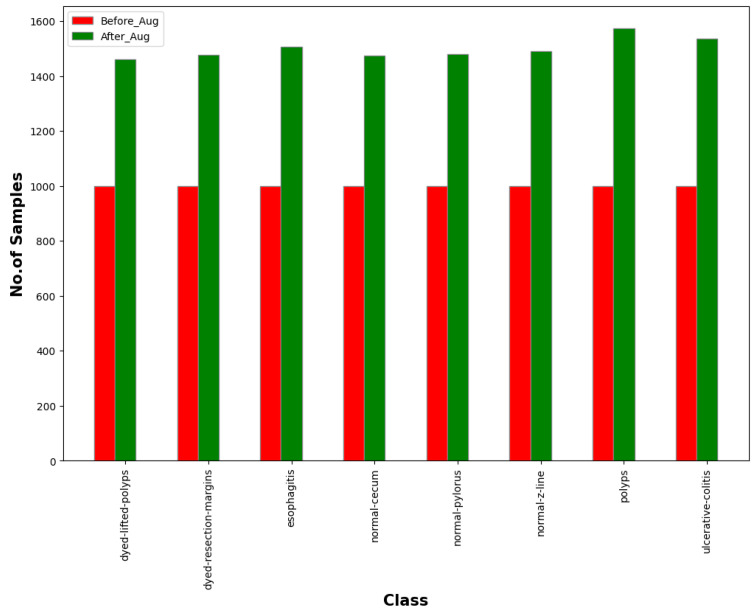
Distribution of samples in each class before and after data augmentation.

Four random geometric transformation techniques such as rotating with a probability of 0.7 with a maximum of 10 left rotation and 10 right rotation, zooming with a probability of 0.3 with a minimum factor of 1.1 and maximum factor of 1.6, and flipping randomly with a factor of 0.2 and top-bottom flip with a factor of 0.8 are used to increase the number of samples in each class. This has resulted in the generation of 12,000 samples from 8000 images. The number of samples for each class before and after augmentation is shown in Figure 2. As Table 1 illustrates, out of the 12,000 images, 9600 were used for training, while the remaining 2400 were used for testing.

## 4. Methods and Techniques

### 4.1. Transfer Learning

Building a robust machine learning/deep learning model in the field of medical research [21] is a challenging task as the number of samples available to build a model is very low. Transfer learning is a good option in such a scenario. Transfer learning is the technique of reusing knowledge gained in one task to perform another similar task [15,22]. Creating a new model from scratch for a small dataset may result in a model with overfitting or a model that may have generalization errors [21]. Sometimes the number of samples per class is not uniformly distributed; in all these scenarios, transfer learning will aid to create a computationally efficient model with less time [23]. In this study, five pre-trained models, namely VGG-16, DenseNet201, InceptionV3, ResNet50, and MobileNet, were used to train the model and achieved validation accuracies of 87.32%, 94.54%, 88.38%, 90.58%, and 76.32%, respectively. The top three pre-trained models selected to create an ensemble model were DenseNet201, InceptionV3, and ResNet50 with respective validation accuracies of 94.54%, 88.38%, and 90.58%. An ablation study was conducted to investigate the effect of pre-trained models on system accuracy, and it was found that the ensemble model [24] created with the three pre-trained models performed better than the other ensemble models, as shown in Table 2.

### 4.2. InceptionV3 Model

**Figure 3:**
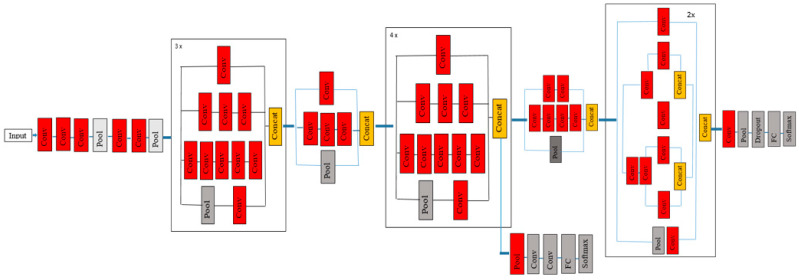
InceptionV3 Model Architecture.

Inception V3 proposed by Szegedy et al. [25] as shown in Figure 3 is a deep neural network with 42 convolutional layers, a pooling layer, and a fully connected (FC) layer. It is mainly used for image classification. It belongs to the Inception family, developed by Google in 2015. When more layers are added to the deep neural network it results in overfitting. To avoid this scenario, in the Inception model, multiple filters with different sizes are used in the same layer. This results in a wide model instead of a deep model. To further improve the accuracy and reduce error rates, four optimization techniques have been added to the Inception V3 model:

- Larger convolution layers are factored into small convolution layers.
- More factorization is performed by adding asymmetric convolutions of the form n × 1.
- Auxiliary classifiers are added to improve the convergence of the network.
- The activation dimensions of the network filters are expanded to reduce the grid size of the model.

### 4.3. ResNet50 Model

**Figure 4:**
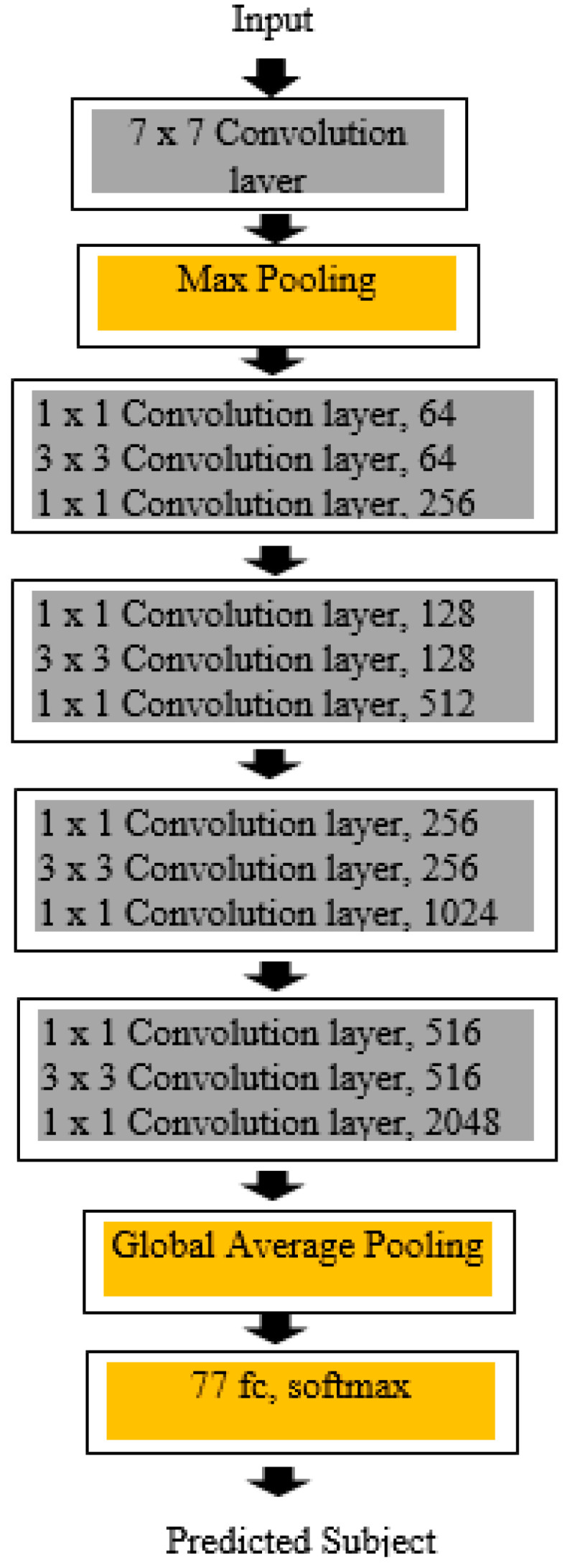
ResNet50 Model Architecture.

**Figure 5:**
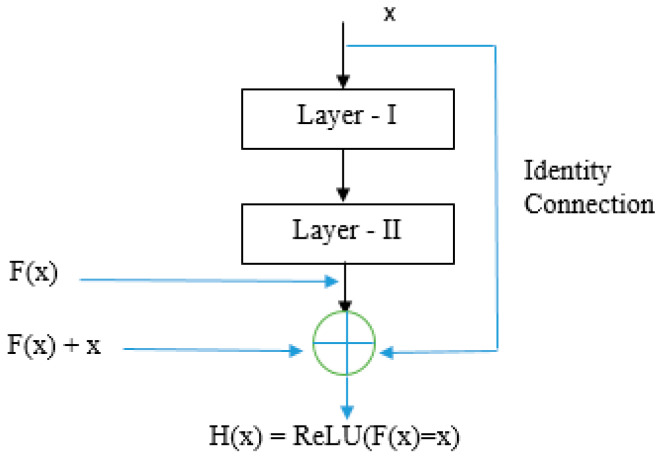
Residual Block used in ResNet-50 architecture.

ResNet50, proposed by He Kaiming et al. [26], has different variants such as ResNet-18, ResNet-34, ResNet50, ResNet101, ResNet110, ResNet152, ResNet164, and ResNet1202. Among the other models, ResNet50 is the most generalized and vibrant. Figure 4 depicts ResNet50, a convolutional network with 50 layers. ResNet-50 is primarily used for image classification and object recognition tasks. As the complexity of the input increases, the neural network model becomes more complex. However, as the number of layers increases, a vanishing gradient problem may arise, and the initial layers may not learn anything during the training phase. To address this problem, ResNet’s skip connection architecture as shown in Figure 5 is used to overcome the vanishing gradient problem.

### 4.4. DenseNet201 Model

**Figure 6:**
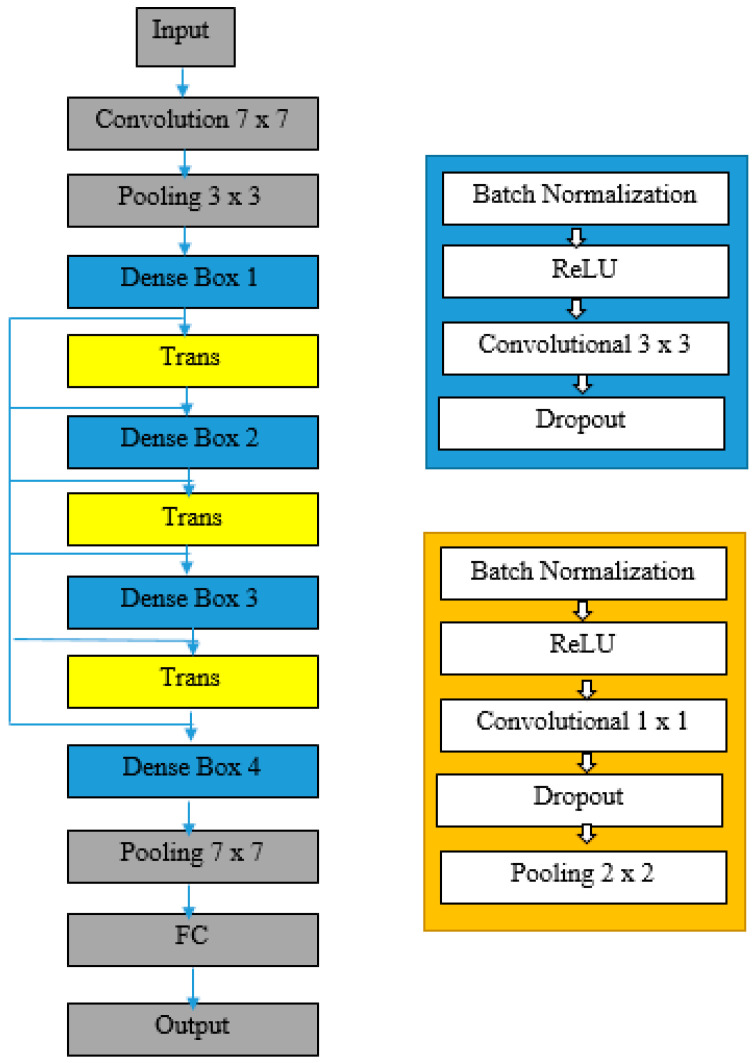
DenseNet Architecture.

DenseNet, proposed by G. Huang [27], is mainly implemented to address vanishing gradient problems such as ResNet. However, DenseNet has few trainable parameters when compared to other convolutional neural network models and hence results in a compact model. In the traditional convolutional model, each layer is sequentially connected to the subsequent layer. In DenseNet, each layer is connected to all preceding layers in the network, resulting in a total of L(L + 1)/2 individual connections among all the layers. DenseNet concatenates the output of the previous layer with the future layer instead of summing it up. Therefore, that feature reuse can be done by eliminating redundant features. Concatenation operation is not possible when the feature map varies in size, and a down-sampling layer is required to reduce the dimensionality of the feature map. This is enabled by dividing the DenseNet into dense blocks as shown in Figure 6. The size of the feature map remains constant within the dense blocks. The transition layers between the blocks consist of three composite operations: batch normalization (BN), a rectified linear unit (ReLU), and a convolution (Conv).

## 5. Proposed Ensemble Model

**Figure 7:**
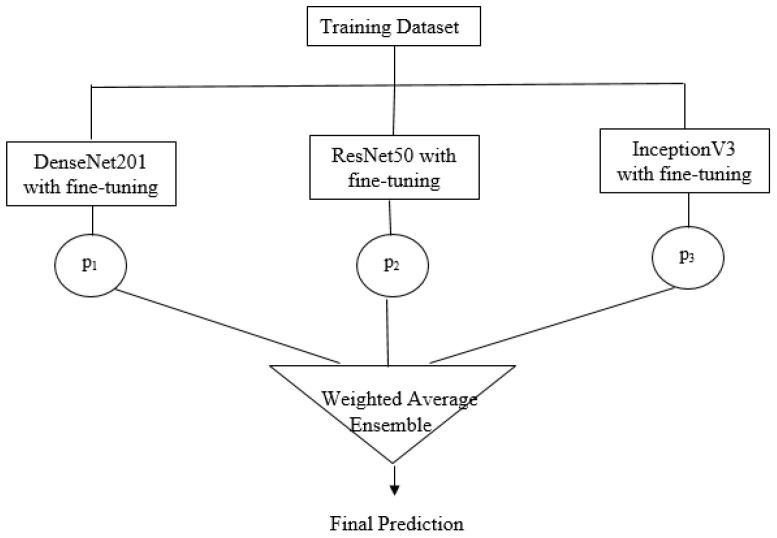
The Architecture of the Proposed Ensemble Model.

Ensemble Models (EM) are used to combine predictions from multiple base models to reduce high variance and bias [28]. In this study, an ensemble model, as shown in Figure 7, has been proposed, consisting of three pre-trained models: DenseNet201, ResNet50, and InceptionV3.

This study uses pre-trained models that have already been trained on the ImageNet dataset. Due to the different number of classes in ImageNet and our dataset, the models are directly loaded from the Keras library without the top layers. Additionally, the weights of the model are downloaded to reduce training time. As the initial layers learn only the basic features from the dataset, they are not trained. Only the top layers are trained to learn the specific features of the dataset. The top layers of the models are replaced with a sequence of Global Average Pooling layers, followed by dense layers with 512, 256, and 128 neurons, respectively, with a ReLU activation function, followed by the Batch Normalization layer and Dropout layer with a value of 0.5. Finally, a fully connected layer is added along with an output layer with eight neurons as there are eight different classes in the KVASIR v2 dataset.

The ensemble model has the advantage of combining the knowledge obtained from the different models. A model may perform well in some classes and poorly in other classes. By merging various models, in ensemble learning, the features that are improperly learned by one model can still be correctly classified by using the pattern learned from another model. There are different methods to create an ensemble model such as:

1. Model Averaging Ensemble;
2. Weighted Averaging Ensemble;
3. Stacking Ensemble, etc.

### 5.1. Model Averaging Ensemble

The model averaging ensemble method [29] is the most followed and simple approach. In this approach, the output of the base learners is averaged to obtain the final prediction of the ensemble model. Due to the high variance and low bias of deep learning architectures, simply averaging the ensemble models improves the generalization performance by bringing down the variance among the models. This is because deep learning models have a tendency to overfit, which means that they have higher training accuracy and lower validation accuracy. As a result, deep learning models do not generalize well to unseen data. This is avoided by averaging the predictions of the multiple base learners. By averaging the predictions, the variance among the models is reduced, leading to accurate generalization performance. The outputs of the base learners are either directly averaged, or the predicted probabilities of the classes are averaged using the SoftMax function. Unweighted averaging is a sensible option when the base learners’ performance is comparable. Since some learners may have lower overall performance but excel at classifying specific subclasses, improving overall performance is possible, and the adaptive meta-learner should be capable of adaptively combining the base learners’ strengths.

The final softmax outputs from all the models were averaged as given in (1).

(1) $$\mathrm{prediction}\;=\;\frac{\sum p_{i}}{N}$$

where *p_i_* is the probability for model *i* and *N* is the total number of models.

### 5.2. Weighted Averaging Ensemble

A weighted ensemble [29,30] is a development of a model-averaging ensemble in which the performance of the model determines the weight of each member’s contribution to the final prediction. The highly-performing model will obtain larger weights than the low-performing model. The mathematical formula to combine the prediction of multiple base learners is given in (2):

(2) $$P(t)=w_{i}p_{i}\left(t\right)$$

where $p_{i}$ is the probability for the model *i*, *N* is the total number of models, and $w_{i}$ is the weight of each model.

### 5.3. Stacking Ensemble

Stacking ensemble models find the most effective way to combine the predictions from two or more base learners using a meta-learning technique. It has a two-layer structure with n-base learners in the first layer and a meta-learner, a linear or non-linear algorithm that combines the predictions of the base learners [31]. The diversity of the base learners and the efficiency with which the base learners’ predictions are merged determine whether the stacking ensemble model is successful. A crucial step in stacking ensemble models is selecting the base learner.

## 6. Experiments

The proposed ensemble model is evaluated in terms of accuracy, precision, recall, and F_1_ score [32,33]. The output of any prediction model can be true or false; in other words, correct prediction or incorrect prediction, respectively. Thus, the classification model can be in any one of the following four states [4]:

- True Positive (TP).
- True Negative (TN).
- False Positive (FP).
- False Negative (FN).

Based on the above classification states, the formula for the different metrics is given below:

(3) $$\mathrm{Accuracy}=\frac{\mathrm{TP}+\mathrm{TN}}{\mathrm{TP}+\mathrm{TN}+\mathrm{FP}+\mathrm{FN}}$$

(4) $$\mathrm{Precision}=\frac{\mathrm{TP}}{\mathrm{TP}+\mathrm{FP}}$$

(5) $$\mathrm{Recall}=\frac{\mathrm{TP}}{\mathrm{TP}+\mathrm{FN}}$$

(6) $$F_{1}\mathrm{Score}=\frac{2\times \;\mathrm{Precision}\;\times \;\mathrm{Recall}}{\mathrm{Precision}+\mathrm{Recall}}$$

Among the actual positive sample, recall (5) is the proposition of the sample that the model has identified as positive. Precision (4) is the proportion of the sample the model has categorized as positive, and F_1_-score (6) is the average of recall and precision [32].

The model was initially trained using pre-trained models such as DenseNet201, InceptionV3, and ResNet50. The model parameters such as the type of optimizer, batch size, no. of epochs trained, the learning rate of the algorithm, training time, and the trainable parameters of each model are given in Table 3.

In this paper, an ensemble model is created with three base learners: DenseNet201 (M1), InceptionV3 (M2), and ResNet50 (M3). The predictions of the base learners are combined using two approaches: model averaging and weighted averaging. In model averaging, all the models are given the same weightage whereas, in the weighted average, the highest-performing model is given more weightage than the low-performing model. The model weights for DenseNet201, InceptionV3, and ResNet50 are set to 0.65, 0.1, and 0.25, respectively, and the validation accuracies are 94.54%, 88.38%, and 90.58%.

**Figure 8:**
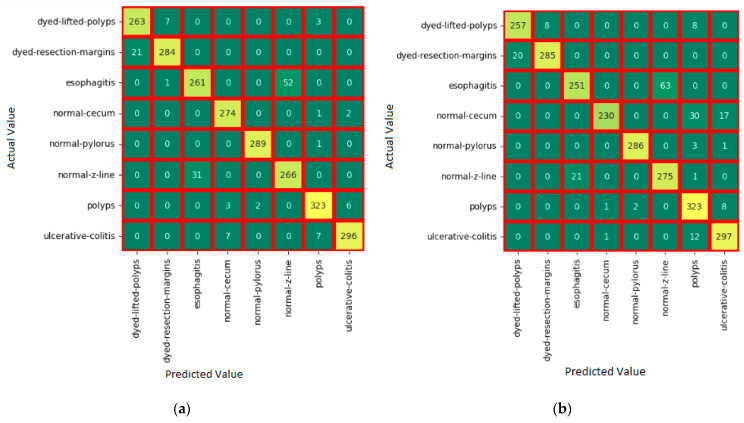

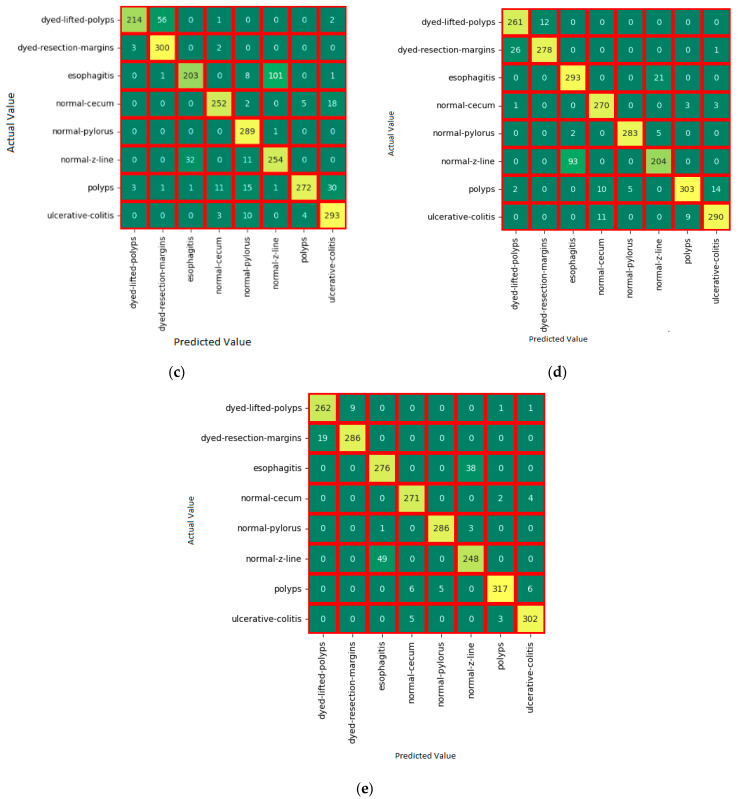
Confusion matrices (CM) of the proposed models. (**a**) Weighted Average Ensemble; (**b**) Model Average Ensemble; (**c**) ResNet50; (**d**) Inception v3; and (**e**) DenseNet201.

**Figure 9:**
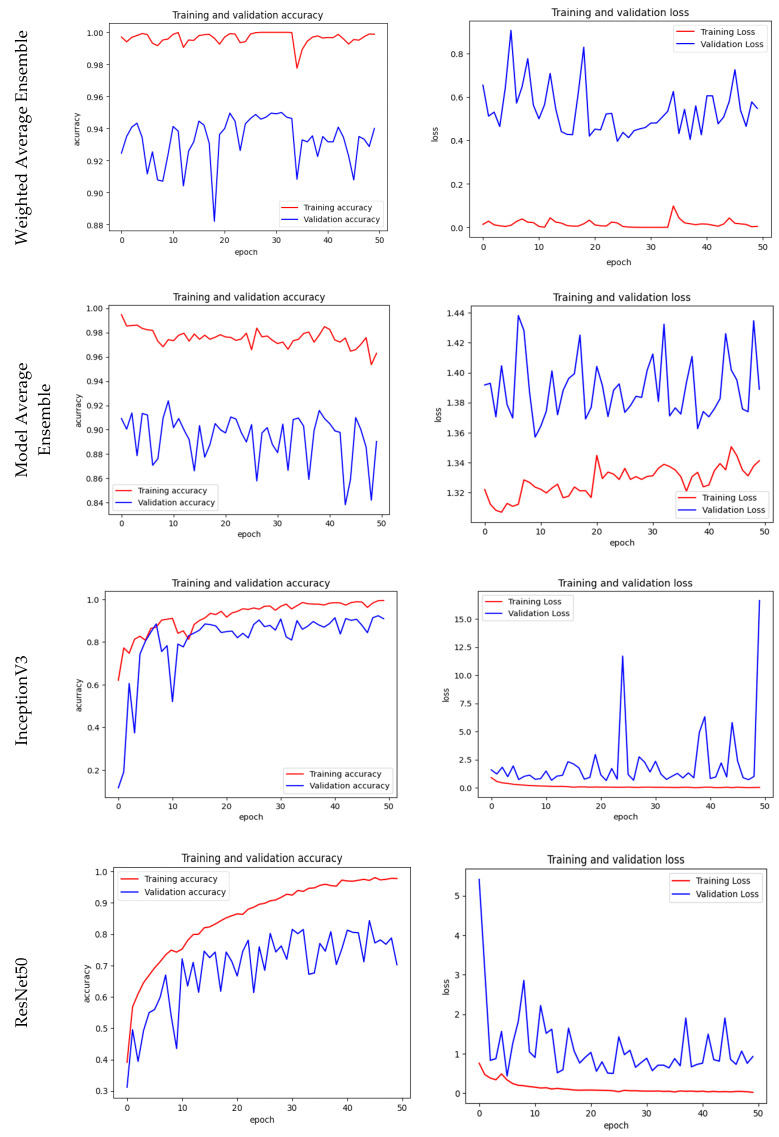

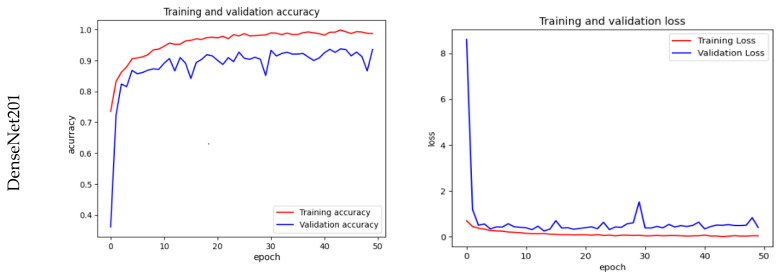
Training & Validation Accuracy and Loss of the proposed models.

The model average and weighted average ensemble models are evaluated on the validation dataset and obtained an accuracy of 92.96% and 95.00%, respectively. The weighted average ensemble model produced more accuracy than the model average ensemble. The confusion matrix for individual pre-trained models and EM models is shown in Figure 8. The number of TP, TN, FP, and FN cases in the validation dataset is shown in the matrix. When compared to other models, the proposed weighted Ensemble model has a higher accuracy of 95.00%. The training and the validation accuracy and loss are given in Figure 9.

### Training, Validation Accuracy & Loss

**Figure 10:**
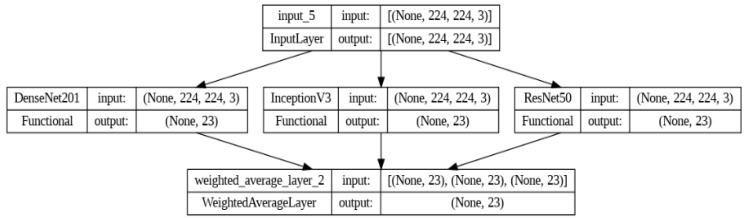
Weighted Average Model Summary.

The experiment was conducted in Google Colab Pro with Python 3 Google Compute Engine backend (GPU–A100) with 40 GB GPU RAM. As previously stated, the individual pre-trained models are loaded, and their lower layers are frozen. Only the top layers are trained to learn the specific feature. Each pre-trained model is trained for 50 epochs in batches of 32 images. The weighted average ensemble model summary is shown in Figure 10. The accuracy of individual models and ensemble models is shown in Table 4. The other performance metrics such as precision, recall, and F_1_ score for the KVASIR v2 dataset are shown in Table 5 and Table 6, respectively.

The proposed weighted average ensemble model is compared with other existing models as shown in Table 7. The AlexNet, GoogleNet, and ResNet50 models reported in reference [1] achieved an accuracy of 97%, 96.70%, and 95%, respectively, but they are trained on the KVASIR dataset with only 5000 images with five classes and no augmentation is performed. The pre-trained model reported in [12] achieved an accuracy of 96.33% but showed signs of overfitting after eight epochs, with accuracy reaching 100%. The model proposed in [13] used a CNN-based spatial attention mechanism to classify the GI tract images and achieved an accuracy of 93.19%. The weighted average ensemble model proposed in [16] achieved an accuracy of 95% using the KVASIR dataset with only 4000 images without data augmentation. The model Dn-CapsNet proposed in [34] achieved an accuracy of 94.16%. Finally, the two-stage classification model proposed in [35] achieved an accuracy of 88% in comparison to all existing models. The weighted average ensemble model proposed in our study demonstrated a better performance for the KVASIR v2 dataset with 8000 samples.

## 7. Discussion

The main objective of this work is to classify the endoscopic images of GI tract diseases. All the existing works have concentrated on creating individual machine learning and deep learning models for classifying endoscopic images. The KVASIR v2 dataset used in this study contains only approximately 1000 samples for each class label. These samples are too few to create a robust model. Therefore, we have used a transfer learning approach to create a model. The ensemble model was created using three pre-trained models: DenseNet201, Resnet50, and InceptionV3. DenseNet201 has the highest validation accuracy of around 95%. We discovered that each model predicts a subset of classes better than the others. As a result, a single model cannot be proposed for this dataset. In this paper, the predictions of the base learners were combined using two techniques, namely model averaging and weighted averaging, that resulted in accuracies of 92.96% and 95%, respectively. The weighted averaging ensemble produced more accuracy than the model average ensemble. Ensemble models have a few drawbacks, including that they require 34% more time for training, a complex architecture (e.g., model averaging and weighted averaging models with 66,941,752 parameters), and an accuracy that relies heavily on the number of base learners and their weights. Furthermore, the accuracy of an ensemble model may vary depending on the dataset’s characteristics. There are several ensemble techniques, including weighted ensemble, bagging, boosting, and stacked ensemble, each with its own set of advantages and disadvantages that are primarily determined by the dataset used. The accuracy of the model can be further improved by segmenting the images before classification.

## 8. Conclusions

In this paper, we proposed an ensemble model called GIT-Net to classify Gastrointestinal tract disorders on the KVASIR v2 dataset. The ensemble model consists of three base learners: DenseNet201, InceptionV3, and ResNet50. We evaluated the performance of the ensemble model using both model averaging and weight averaging methods. Model averaging treats all models equally, resulting in an accuracy of 92.96%. Alternatively, a weighted average ensemble assigns higher weights to models with higher accuracy, resulting in an accuracy of 95.00%, which is higher than the model averaging approach. Therefore, weighted average ensemble models perform much better than the individual pre-trained models.

## Figures and Tables

**Table 1:** Dataset Details.

	KVASIR v2 Dataset
No. of Samples	8000
No. of Classes	8
No. of Samples after Augmentation	12,000
Training Dataset	9600
Testing Dataset	2400

**Table 2:** Performance Measure on Number of Base-Learners.

Ensemble Model	Accuracy
ResNet50 + InceptionV3	90.32
InceptionV3 + DenseNet201	87.00
ResNet50 + DenseNet201	89.43
DenseNet201 + InceptionV3 + ResNet201	95.00

**Table 3:** Parameters of Model Architecture.

Options	DenseNet201	InceptionV3	ResNet50	Average Ensemble	Weighted Average Ensemble
Optimizer	Adam	Adam	Adam	Adam	Adam
Batch Size	32	32	32	32	32
Epochs	50	50	50	50	50
Learning Rate	0.0001	0.0001	0.0001	0.0001	0.0001
Training Time	39 m 76 s	17 m 41 s	14 m 34 s	68 m 73 s	69 m 95 s
Trainable Parameters	19,223,880	22,978,472	24,739,400	66,941,752	66,941,752
No. of features extracted	8	8	8	Nil	Nil

**Table 4:** Performance Evaluation on KVASIR v2 Dataset.

Model	KVASIR v2 Dataset Accuracy
DenseNet201 (M1)	94.54
InceptionV3(M2)	88.38
ResNet50 (M3)	90.58
Model Averaging Ensemble	92.96
Weighted Average Ensemble	95.00

**Table 5:** Performance Evaluation of Pre-trained Models.

Class	Precision			Recall			F_l_-Score		
	Ml	M2	M3	Ml	M2	M3	Ml	M2	M3
dyed-lifted-polyps	95.70	92.00	97.52	95.67	92.60	78.70	95.19	92.72	87.27
dyed-resection-margins	96.01	96.86	84.76	95.75	93.15	98.36	96.38	95.45	90.59
esophagitis	93.98	79.52	86.97	83.81	82.31	65.93	88.85	80.48	74.35
Normal-cecum	97.11	96.78	94.12	99.19	90.47	91.78	98.14	93.07	92.95
normal-pylorus	98.32	97.26	86.29	99.31	82.59	100.00	98.79	89.92	92.12
normal-z-line	84.42	73.70	71.96	93.85	86.69	86.60	88.60	79.42	78.86
polyps	98.22	91.16	97.98	96.51	88.72	81.22	97.37	89.37	88.58
ulcerative-colitis	96.32	90.19	85.64	98.87	94.55	98.94	97.57	92.85	90.79

**Table 6:** Performance Evaluation of Ensemble Models on KVASIR v2 Dataset.

Class	Precision		Recall		F_l_-Score	
	Model Average Ensemble	Weighted Average Ensemble	Model Average Ensemble	Weighted Average Ensemble	Model Average Ensemble	Model Average Ensemble
dyed-lifted-polyps	93.52	93.00	94.70	96.85	93.27	94.10
dyed-resection-margins	97.76	97.96	93.36	93.45	95.59	95.12
Esophagitis	92.97	89.78	80.93	83.44	86.35	86.88
Normal-cecum	99.12	96.45	83.78	99.88	90.95	98.65
normal-pylorus	99.29	99.12	99.97	100.00	99.12	99.45
normal-z-line	81.96	84.11	93.97	90.78	87.89	87.77
Polyps	86.98	96.32	97.60	97.12	91.58	97.64
ulcerative-colitis	92.64	97.89	96.22	95.78	94.79	96.33

**Table 7:** Comparison of proposed model with other recent models.

Previous Studies	Model	Accuracy	Dataset Samples	Augmentation
Mosleh [1]	AlexNet	97.00%	5000 images with 5 classes	Not done
	GoogleNet	96.70%		
	ResNet50	95.00%		
YogaPriya [12]	Transfer Learning	96.33%	5000 images	Done
Zenebe [13]	CNN based on Spacial attention Mechanism	93.19%	KVASIR v2 with 8000 images	Done
Muhammed [16]	Weighted Avg	95.00%	KVASIR with 4000 images	Done
Afriyie et al. [34]	Dn-CapsNet	94.16%	KVASIR v2 with 5000 images	Not done
Pozdeev et al. [35]	Two Stage Classification	88.00%	KVASIR v2 with 8000 images	Done
Proposed Weighted Average Ensemble		95.00%	KVASIR v2 with 8000 images	Done

## Data Availability

The data used for this study is available at https://www.kaggle.com/datasets/plhalvorsen/KVASIR-v2-a-gastrointestinal-tract-dataset (accessed on 3 January 2023).
